# Editorial: Molecular mechanisms of dendritic spine pathology in neurodevelopmental and psychiatric disorders

**DOI:** 10.3389/fnmol.2023.1159451

**Published:** 2023-02-21

**Authors:** Karun Kar Singh, Sehyoun Yoon

**Affiliations:** ^1^University Health Network (UHN), Faculty of Medicine, University of Toronto, Toronto, ON, Canada; ^2^Department of Neuroscience, Northwestern University, Chicago, IL, United States

**Keywords:** neurodevelopment, dendritic spine, plasticity, molecular mechanism, mouse model

Dendritic spines undergo morphological changes in response to experiences and stimuli, and subtle changes in dendritic spines can significantly affect synaptic function, connectivity patterns, and plasticity of neural circuits. Disruption of synaptic circuitry affects cognitive, social, and emotional processing, and is associated with neurodevelopmental and psychiatric disorders. Numerous genes have been identified that play a role in synaptic regulation. Studies of mechanisms regulating synaptic growth and structural plasticity are critical in understanding the etiology of these disorders.

This Research Topic covers a large spectrum of neuropathology, genetics, molecular mechanisms, and animal model studies investigating the etiology of neurodevelopmental and psychiatric disorders.

Sungur et al. focused on the role and function of profilin1, a regulator of the actin cytoskeleton and a critical factor in determining the structure of the dendritic spine. Although no change in spine density and morphology was observed in the previously reported adult mouse lacking profilin1 (Görlich et al., 2012), the authors confirmed a critical time point by analysis of dendritic spine density in the hippocampal CA1 region of the brain-specific profilin1 knockout mouse model. In addition, reduced social and object recognition and ultrasonic vocalizations were observed in juvenile conditional knockout mice. These results suggest a potential pathological mechanism whereby the change in spine density in the brain during the juvenile period, caused by G-actin deficiency, causally affects cognitive impairment and social communication.

For a better understanding of multi factors, Ford et al. discuss the role of ARID1B, KANSL1, and WDR5 in neural connectivity, dendritic spine, and synapse development, and neurological behaviors. They also associated cellular and molecular signaling, which provides potential therapeutic targets for chromatin modifier-associated autism spectrum disorders and intellectual disability. The authors summarized representative papers on these proteins, including recent studies using knockout mouse models and induced pluripotent stem cells derived from patients. They discussed therapeutic strategies focusing on the dendritic spine and synaptic pathology. Also, Shimada and Yamagata have covered various aspects of tuberous sclerosis complex (TSC) (due to mutations in the *Tsc1* or *Tsc2* genes), including dendritic spine plasticity and neurological and behavioral impairment. This exciting review suggests that the well-defined Tsc1/2-induced regulation of Rheb1 activation, and Rheb1-induced mTOR and Syntenin regulation mechanisms, could be associated with spine development and synaptic function. Although more elaborate follow-up studies are needed, it should be noted as a target mechanism for recovering TSC-related neurodevelopmental disorders (epilepsy, intellectual disability, and autism).

Finally, the review article by Zaccard et al. focused on the mechanisms of spinule formation and its function and implications for neurodevelopmental disorders and psychiatric diseases. Dendritic spinules are microstructures protruding from the spine membrane, and they are difficult to observe due to their structural fragility and small size in neurons. Along with introducing the research methods to overcome these difficulties over 80 years, the authors elucidated the molecular mechanism of spinule formation and its effect on synaptic plasticity and various diseases.

Overall, we hope that this Research Topic, which addresses the factors and mechanisms that regulate synaptic structure in the brain, will be helpful to researchers interested in understanding the pathology of neurodevelopmental and neuropsychiatric disorders, and suggesting potential therapeutic targets.

## Author contributions

All authors listed have made a substantial, direct, and intellectual contribution to the work and approved it for publication.
